# Cultural adaptation of the Safety Attitudes Questionnaire – Short Form (SAQ-SF) in Poland

**DOI:** 10.1371/journal.pone.0246340

**Published:** 2021-02-05

**Authors:** Iwona Malinowska-Lipień, Piotr Brzyski, Teresa Gabryś, Agnieszka Gniadek, Maria Kózka, Paweł Kawalec, Tomasz Brzostek, Allison Squires

**Affiliations:** 1 Institute of Nursing and Midwifery, Faculty of Health Sciences, Jagiellonian University–Medical College, Krakow, Poland; 2 Dziupla Statistical Analysis, Warsaw, Poland; 3 Institute of Public Health, Faculty of Health Sciences, Jagiellonian University–Medical College, Krakow, Poland; 4 Rory Meyers College of Nursing, New York University, New York, New York, United States of America; Florida International University, UNITED STATES

## Abstract

**Background:**

It is essential to provide safe healthcare in complex, difficult, and quickly changing conditions. The quality of healthcare services directly influences the safety of both the patients and staff. Understanding healthcare staff attitudes toward safety in the healthcare delivery context is foundational for building a culture of safety.

**Aim of the work:**

To adapt, via a structured translation methodology, the Safety Attitudes Questionnaire–Short Form (SAQ-SF), which assesses how employees of the health care sector perceive the safety climate in their workplace, to the Polish context.

**Methods:**

Using a content validation approach to structure the translation process, we tested and psychometrically analysed the translated SAQ-SF. The sample comprised 322 employees of a district hospital (second referral level, which ensures 24/7 emergency care services) in Poland.

**Results:**

The reliability of the sub-scales of the Polish version of the SAQ-SF ranged from 0.66 to 0.95. The discriminatory power of particular SAQ items ranged between 0.02 and 0.90. For 6 out of the 8 scale dimensions, the questions with the highest factor loadings were those measuring the same dimensions of the safety climate, according to the original scale.

**Conclusions:**

The Polish version of the SAQ-SF (SAQ-SF-PL) meets the criteria of psychometric and functional validation as well as demonstrates good reliability as a measure of patient safety culture in the Polish context. The SAQ-SF-PL is an instrument that enable a valid and reliable assessment of patient safety climate in the Polish healthcare facilities and identify opportunities for improvement. International comparisons will also become easier.

## Introduction

Scientific evidence shows a positive correlation between safety culture, safety climate, and work safety [1–3]. Managers are key actors in setting the overall working climate and their decisions influence the health and life of both the patient and employee [4, 5]. In healthcare, a safety climate is particularly important owing to the specific nature of healthcare facilities and associated responsibilities of workers to protect patients from harm when receiving their services. According to Pronovost et al., safety-oriented activities and actions in healthcare facilitates are undertaken generally when adverse events are taking place or medical errors have occurred. Therefore, there is a need for a more comprehensive analysis, which would help detect the real causes of those events before they happen, and which would facilitate development of targeted preventive measures [6]. In everyday clinical practice, the term "safety climate" may sound more suitable, as it generally refers to measurable components of safety culture, such as activities within management, safety systems and perception of safety by employees [7, 8].

In 2009, the Council of the European Union issued its recommendations on patients’ safety. The EU based the recommendations on the patient safety research from the World Health Organization (WHO) through the World Alliance for Patient Safety, as well as by the Organisation for Economic Co-operation and Development. The EU Council’s recommendations stated that without necessary processes and policies to prevent medical errors, threats to patient safety pose a serious health problem and generate a significant economic burden. It further concluded that adverse events directly resulting from the absense of patient safety infrastructure could be largely avoided in any care delivery context. The Council’s report also indicated that system factors are responsible for most adverse events and threats to patients. Consequently, the Council of the European Union recommended to healthcare organizations that they: 1) consider the patients’ safety as a priority issue in the policy and projects on health on the country, regional and local levels; 2) regularly check and update safety standards or exemplary solutions applicable in the healthcare on the territory of a particular member state; and 3) mobilise organisations of medical staff to play an active role in building up patients’ Safety Climate [9, 10].

In Poland, there is a growing concern to improve patients’ safety in healthcare facilities [11]. Few studies have addressed the topic, while their findings are similar to the international literature [12]. A common barrier to assessing patient safety culture is the lack of a reliable and valid Polish translation of instruments designed to assess various dimensions of patient safety and link those findings to outcomes. Therefore, the aim of the present study was to culturally adapt the Safety Attitudes Questionnaire–Short Form (SAQ-SF) to the Polish setting and pilot test it using a cross-sectional design. The SAQ-SF is a widely used instrument for assessing the attitudes of employees of the healthcare sector towards the issue of workplace and patients’ safety [13]. Numerous studies have indicated that the SAQ offers good psychometric properties [13–27]. Translations into thirteen languages suggest the psychometric properties are stable, including: Albanian [14], Arabic [15], Danish [16], Chinese [17, 18], Croatian [19]; Dutch [20], German [21], Italian [22], Norwegian [23], Portuguese [24], Slovenian [25], Swedish [26] and Turkish [27]. This study will help ensure that a reliable and valid translation of the instrument, capable of generating internationally comparable data is available for Polish healthcare facilities.

A variety of different formal regulations, procedures, standards and guidelines are being formulated and implemented, with the goal of creating a safety culture in every Polish healthcare organization. Despite numerous policy actions, medical staffs do not always act according to the regulations. While safety culture is frequently debated in Poland, reports from safety assessments in healthcare facilities, especially hospitals, are still missing. The SAQ-PL questionnaire is expected to allow of patient safety climate assessment across the medical sector in Poland.

### Aim of the work

The research focuses on how to adapt, via a structured translation methodology, the Safety Attitudes Questionnaire–Short Form (SAQ-SF), which assesses how employees of the healthcare sector perceive the safety climate in their workplace, to the Polish context.

## Material and methods

### Study group

#### Design

This was a cross-sectional study which sought to evaluate psychometric properties of the Polish language translation of the SAQ-SF. The procedure of the SAQ questionnaire validation was approved by the Center for Healthcare Quality and Safety Team of the University of Texas at Houston-Memorial Hermann. The research was also approved by the Bioethics Committee of Jagiellonian University No. 122.6120.286.2016.

Written consent for participation was obtained prior to data collection. The privacy and confidentiality of participants was strictly protected as follows: (1) all the information provided by each participant was coded by a number that does not directly identify any individual; (2) all identifying information was coded and removed from all non-numerical data to make it impossible for anyone but the experimenter to identify any individual.

#### Patient and public involvement

Concerns about widely understood patient safety in Poland, both from healthcare professionals and public reports, were the reasons for adapting the instrument to the Polish setting. While patients were not involved in the recruitment or design of this study, health care professionals were, and many of them have friends and relatives who are or were, patients in the Polish system. The translated instrument will be widely shared with Polish facilities so they can conduct their own internal organisational assessments and improve patient safety based on their needs.

#### Description of the instrument

The SAQ was developed by Sexton et al. in 2006 via a multistep process, and it was validated using the explorative and confirmatory factor analysis (CFA) on the data from 203 clinical areas in the United States, the United Kingdom, and New Zealand. The exploratory factor analysis was applied to explore the existence of a latent structure of the items, yielding six factors. Psychometric properties of this instrument have been analyzed and reported, showing evidence of validity and reliability [13]. We used the SAQ-SF, an instrument that assesses how employees of the healthcare sector perceive the issues of safety climate [13]. The two-part questionnaire contains 41 items. One part contains demographic questions about the participant’s gender, work position, professional experience, patient age group being served at work, i.e. children, adults, or both groups [13]. The main assessment includes 36 questions and is sub-divided into 6 safety-related climate scales (Table 1). The first part also contains additional five questions not included in any of the above mentioned sub-scales, i.e. question 14 referring to the assessment of the managing person for safety provision, and questions from 33 to 36 concerning the assessment of conflicts and cooperation between members of an inter-disciplinary team (e.g. nurses, doctors and pharmacists). Participants respond using a 5-point Likert scale (1 = strongly disagree (A); 2 = disagree (B); 3 = neutral (C); 4 = agree (D) and 5 = strongly agree (E)), and for each question of the form the authors included also the option “does not concern” [13]. When calculating the results, a conversion to 100-point scale (i.e.: 1 = 0; 2 = 25; 3 = 50; 4 = 75; 5 = 100) is used. In questions 2, 11 and 36 a reverse scale is used, which has to be considered during calculations and interpretations. The final result is between 0 and 100, where zero means the worst, and 100 is the best perception of safety climate. Results which equal 75 or beyond are considered positive [13]. The percentage of respondents who are positive (i.e. percent of compliance) indicates respondents who received a score of 75 or higher (agree or strongly agree).

**Table 1:** SAQ subscale and definition.

SAQ Subscale	Questions	Definition
Teamwork Climate	from 1 to 6	assesses the perception of the quality of cooperation between employees
Safety Climate	from 7 to 13	assesses the perception of organisational engagement of employees for safety
Job Satisfaction	from 15 to 19	assesses subjective positive feelings related with professional experience
Stress Recognition	from 20 to 23	assesses the influence of stress factors on the work efficiency
Perception of management	from 24 to 28	assesses management staff at the level of a ward and a hospital,
Work conditions	from 29 to 32	assesses the perception of the quality of environmental and logistic support at workplace (e.g. equipment, facilities and professionals)

Notes: The main assessment includes 36 questions and is sub-divided into 6 safety-related climate scales. Five questions not included in any of the above mentioned sub-scales: 14,33–36.

#### Approach to the adaptation process

The equivalence between the adapted tool and the original version was assessed in five aspects: graphic layout (e.g. appearance of the test and instructions, the manner of calculating the results), translation (the content of the questions, the level of difficulty of wording), functional (usefulness for the same purpose), reconstruction (methods of checking its reliability and validity, research procedure, types of norms) and psychometric (reliability, correlation between versions, correlation with other scales) [28–31]. The SAQ-SF may be used in various contexts of the healthcare system, which is why not only technical and semantic equivalence of the items were assessed, but also the meaning of the items in relation to the specifics of work in the Polish healthcare sector [28–31].

We then undertook a five-stage adaptation process of the SAQ-SF. First, we obtained permission to use the questionnaire from the authors of the original version (direct contact with the authors of the questionnaire was necessary). The next step was to translate the original version of the questionnaire from English to Polish independently by two translators. They were both Polish native speakers with a degree in English Philology and were also experienced translators. Two independent translations of the tool were then used to obtain an initial version of the Polish version of the questionnaire. Then the project manager reviewed all items of the questionnaire and identified potentially problematic questions, phrases or terms that might cause difficulty or ambiguity in the translation from English into Polish. The next stage was the backward translation, i.e. translation of the newly obtained version into the original language by a translator whose mother tongue was English and who was fluent in Polish. Afterwards, a group of five bilingual researchers was asked for their opinions on the comparability of the translated text with the original scale of SAQ-SF version. It was the first level of an overview made by the panel of experts. At this stage, a team assessment of the questionnaire was conducted. Necessary changes were introduced and the Polish version of the questionnaire was verified by English-speaking translators to confirm that the meaning of the original questionnaire remained unchanged.

We then replicated the process described by Squires et al. [30] with some modifications. Differences in our approach included a larger number than of 17 experts [31, 32], comprising 6 practising nurses, 2 nurses with scientific educational background, 3 physicians, 3 physiotherapists and 3 paramedics. The experts were fluent in Polish and English and had professional experience within the last five years working in the Polish healthcare system. The experts assessed the questions in the online version of the questionnaire with each expert receiving a unique link to the questionnaire. Each question was assessed for its content, i.e. the meaning of the translation, and conceptual meaning, i.e. relation to the Polish healthcare system (the scale of the assessment referring to all the questions: 1 = does not concern, 2 = slightly significant, 3 = very significant) [30]. Moreover, for every item they were asked to answer the “yes/no” questions of whether the translation was semantically and technically equivalent to the original question. Then the research team performed an analysis of the received feedback, and on the basis of the feedback the wording of some questions was verified so as to ensure intercultural consistency and possibility of application in Poland. The accuracy of the final translation was assessed and Cronbach’s alpha coefficient was 0.98. Then, the validation study of the questionnaire SAQ-SF was conducted on a sample of hospital staff.

#### Sample & setting

The validation study of the questionnaire SAQ-SF was conducted on a sample of hospital staff. The study group included the employees of one of hospitals (second referral level, which ensures 24/7 emergency care services) in Poland, which employed 740 people, including nurses, physicians, physiotherapists, paramedics, medical carers and administrative staff. All indicated Polish nationality.

At the hospital, a hospital coordinator was appointed to liaise with the research team and ensure consistency in data collection in line with the study guidelines. In clinical departments, the questionnaire was distributed during departmental staff meetings led by the hospital project coordinator. Participants had 4 weeks to complete the questionnaire. The completed questionnaires were thrown into a closed box with a hole. After 4 weeks, the coordinator was collected the boxes, secured them and handed them over to the research team. Participants were informed that participation was voluntary and anonymous, all responses would be kept confidential and no individual responses would be available to the hospital management. The inclusion criterion was informed consent to participate in the study and Polish nationality. The exclusion criterion was the lack of consent and personnel of nationality other than Polish.

#### Data collection

All the employees of the test site were invited to participate in the study, of whom 474 (64.0%) completed and returned the questionnaire. The final analysis included the results of 322 respondents (43.5%) who provided complete data. People who indicated Polish nationality and filled out the entire questionnaire were included in the research. Therefore, based on these inclusion criteria, 152 participants were excluded from the final sample. The study was conducted between March and June 2018.

### Statistical analysis

The theoretical validity of the scale was assessed by the principal components analysis (PCA) with Varimax rotation. Only questions assigned to one of the 6 sub-scales of the questionnaire SAQ-SF were analysed. Questions not assigned to any of the sub-scales, i.e. questions 14, 33–36, were excluded from the analysis. As a selection criterion for the number of extracted components, the Kaiser criterion of eigen value higher than 1 and the interpretation of the meaning of the results were used. It was expected that the items of the particular sub-scales would have respectively high (>0.6) factor loadings on the same components. To assess the adequacy of implementing a factor analysis for the analysed set of questions the Kaiser-Mayer-Olkin (KMO) coefficient was used [33]. Its values range from 0 to 1, and higher values of the coefficient indicate that if the analysed set of variables is subjected to the factor analysis it will give a chance to obtain well-interpretable factors.

Reliability in the aspect of internal consistency was evaluated using the Cronbach’s alpha coefficient; the values of the coefficient above 0.7 were deemed satisfying, and over 0.8 as good. The change of the alpha coefficient after removing the item from the scale was analysed as well, and the discriminatory power of the scale item calculated as correlation of particular items with the total score of the scale to which a particular item belongs after removing this item from the total score. The correlation was expected to be at least 0.3 [34].

## Results

### Characteristics of the researched group

The study group contained 80.4% women. The sample’s professional breakdown included 24.8% Physicians, 58.4% Nurses (including 23.6% with a masters or higher degree) and the remaining were other representatives of health professions. A majority of the staff had been working in the hospital for over 5 years (62.4%). Complete participant descriptive statistics are found in Table 2.

**Table 2:** Participant demographics (N = 322).

Variables		n	%
**Sex:**	Female	259	80.4
	Male	63	19.6
**Possition:**	Nurse /Nurse Practitioner	188	58.4
	Physician (Staff Physician; Fellow Physician)	80	24.8
	Missing data	21	6.5
	Other (Environmental Support, Clerk/ Secretary/ Receptionist)	13	4.0
	Therapist	10	3.1
	Technologist/Technician	10	3.1
**Primary working place:**	Only adults ward	144	44.7
	Both on adult or pediatric wards	107	33.2
	Only pediatric ward	50	15.5
	Missing data	21	6.5
**Years of professional experience:**	1 to 5 years	93	28.8
	>20 years	75	23.3
	6 to 10 years	69	21.4
	11 to 20 years	57	17.7
	<1 year	24	7.4
	Missing data	4	1.2

Notes: Complete participant descriptive.

### Validity

Before performing the analysis validity of the Polish adaptation of SAQ-SF, the Kaiser test was used to check whether the data meet the requirements of the factor analysis. The KMO value, being the measure of the adequacy of the sample selection, was estimated at the level of 0.87 (df = 8630, p<0.001). The PCA extracted 9 components with eigen values higher than 1. However, the analysis of the solutions with another number of factors revealed the 8-component model was the closest to the original factor structure of the tool and allowed the clearest interpretation of the factors. This model explained 68% of the total variance of the analysed set of variables.

Varimax rotation demonstrated that on the first and second factors, variables describing the management at hospital or at ward level respectively had high loadings. On the third factor, high loading was revealed for variables describing the Safety Climate and the Teamwork Climate. Not all variables contained in the original version of SAQ-SF in the dimension of Teamwork Climate possess their highest loads on the third factor. Item No. 6 (The physicians and nurses here work together as a well-coordinated team.) and question No. 1 (Nurse input is well received in this clinical area) had their highest factor loadings on the 7^th^ component. On the very same component the item 3 (Disagreements in this clinical area are resolved appropriately (i.e., not who is right, but what is best for the patient), the 3^rd^ component, had a slightly lower factor loadings than on the 1^st^ component. The variables evaluating Job Satisfaction had the highest loads on the 4^th^ component. The variables describing Stress Recognition had the high loads on the 5^th^ component, while on the 6^th^ component variables defining the sub-scale evaluating the Working Conditions had high loadings. On the 7^th^ component, the highest factor loadings belong to the two variables from the dimension of the team work climate. The 8^th^ component included groups variables (questions No. 2 and No. 11) that are statements presented in the reverse form (Table 3), and according to the original structure of the scale they belonged to the domain of Teamwork Climate and Safety Climate.

**Table 3:** The values of factor loadings of statements of the Polish version of SAQ-SF, obtained with exploratory factor analysis (N = 322).

		Component							
		1	2	3	4	5	6	7	8
**Percent of the explained total variance after rotation**		12.4	11.9	10.1	9.2	8.1	7.0	5.3	4.0
**Teamwork Climate**	1. Nurse input is well received in this clinical area.							0.714	
	2. In this clinical area. it is difficult to speak up if I perceive a problem with patient care.								0.662
	3. Disagreements in this clinical area are resolved appropriately (i.e. not *who* is right. but *what* is best for the patient).	0.340		0.531				0.310	
	4. I have the support I need from other personnel to care for patients.			0.616					
	5. It is easy for personnel here to ask questions when there is something that they do not understand.			0.730					
	6. The physicians and nurses here work together as a well-coordinated team.				0.359			0.573	
**Safety Climate**	7. I would feel safe being treated here as a patient.		0.319	0.491				0.351	
	8. Medical errors are handled appropriately in this clinical area.		0.315	0.592				0.336	
	9. I know the proper channels to direct questions regarding patient safety in this clinical area.			0.660	0.338				
	10. I receive appropriate feedback about my performance.			0.656					
	11. In this clinical area. it is difficult to discuss errors.								0.858
	12. I am encouraged by my colleagues to report any patient safety concerns I may have.	0.315		0.382					
	13. The culture in this clinical area makes it easy to learn from the errors of others.			0.398	0.374				
**Job Satisfaction**	15. I like my job.				0.626				
	16. Working here is like being part of a large family.				0.717				
	17. This is a good place to work.				0.776				
	18. I am proud to work in this clinical area.			0.311	0.715				
	19. Morale in this clinical area is high.				0.614				
**Stress Recognition**	20. When my workload becomes excessive. my performance is impaired.					0.840			
	21. I am less effective at work when fatigued.					0.863			
	22. I am more likely to make errors in tense or hostile situations.					0.821		-0.340	
	23. Fatigue impairs my performance during emergency situations (e.g. emergency resuscitation. seizure).					0.789			
**Perceptions of Management**	24. Management supports my daily efforts: Unit /Mgt	0.859							
	24. Management supports my daily efforts: Hosp /Mgt		0.810						
	25. Management doesn’t knowingly compromise pt safety: Unit/ Mgt	0.837							
	25. Management doesn’t knowingly compromise pt safety: Hosp/ Mgt		0.789						
	26. Management is doing a good job: Unit /Mgt		0.880						
	26. Management is doing a good job: Hosp /Mgt	0.904							
	27. Problem personnel are dealt with constructively by our: Unit /Mgt	0.879							
	27. Problem personnel are dealt with constructively by our: Hosp /Mgt		0.841						
	28. I get adequate. timely info about events that might affect my work. from: Unit /Mgt	0.880							
	28. I get adequate. timely info about events that might affect my work. from: Hosp /Mgt		0.843						
**Working Conditions**	29. The levels of staffing in this clinical area are sufficient to handle the number of patients.						0.443		
	30. This hospital does a good job of training new personnel.						0.753		
	31. All the necessary information for diagnostic and therapeutic decisions is routinely available to me.						0.692		
	32. Trainees in my discipline are adequately supervised.						0.753		

* Shaded columns indicate the highest factor loading of variables, which within individual components belong to the same dimension of the SAQ scale.

** Hosp–hospital; Mgt-management, Pt- patient.

**Notes:** Varimax rotation demonstrated that on the first and second factors, variables describing the management at hospital or at ward level respectively had high loadings. On the third factor, high loading was revealed for variables describing the Safety Climate and the Teamwork Climate.

### Reliability

The Cronbach’s alpha coefficient for the domain of Teamwork Climate amounted to 0.66. After removing the item 2 (question No 2 –“In this clinical area, it is difficult to speak up if I perceive a problem with patient care”) the reliability of the sub-scale increased to 0.69. In the case of the Safety Climate domain, reliability was 0.74 and increased only after removing the item 11 (In this clinical area, it is difficult to discuss errors) to the level of 0.82. For the Job Satisfaction, reliability reached 0.82, and only after removing item 15 (No 15—I like my job) the value of the reliability coefficient increased to 0.84. The value of the alpha coefficient for the sub-scale of Stress Recognition was estimated as 0.86. For the domain of Working Conditions the value of the coefficient was 0.75 and increased to the level of 0.80 after removing item 29 (The levels of staffing in this clinical area are sufficient to handle the number of patients). The domain referring to the Perceptions of the Management refers separately to the supervisors of a particular ward and the management of the hospital respectively. The alpha coefficient for Perceptions of Management at the unit level was 0.95 whereas at the hospital level– 0.93. Removal of any item did not increase the value of the coefficient for this scale either at the unit or the hospital level.

The discriminatory power of particular entries of SAQ-SF scales ranged from 0.02 for item 11 (In this clinical area, it is difficult to discuss errors) to 0.90 for item 26 (Management is doing a good job). The discriminatory power of the Teamwork Climate scale ranged from 0.22 for item 2 (In this ward it is difficult to talk openly if I notice a problem concerning patient care) to 0.54 for item 5 (It is easy for personnel here to ask questions when there is something that they do not understand). The discriminatory power of the Safety Climate scale ranged from 0.02 for item 11 to 0.64 for item No. 8 (In this ward issues concerning adverse events are handled correctly). The discriminatory power of the Job Satisfaction scale ranged from 0.37 for item 15 (I like my job) to 0.73 for item No. 18 (I am proud to work in this clinical area). The discriminatory power of the Stress Recognition scale items ranged from 0.65 for item 23 (Fatigue impairs my performance during emergency situations (e.g. emergency resuscitation, seizure) to 0.75 for question No. 21 (I am less effective at work when fatigued)). The discriminatory power of the sub-scale item measuring the perception of the management scale at the unit level ranged from 0.73 for item No. 25 (Management doesn’t knowingly compromise patient safety) to 0.90 for item No. 24 (Management supports my daily efforts). For items relating to hospital management, the discriminatory power ranged from 0.76 to 0.88. Finally, the discriminatory power of the Working Conditions scale ranged from 0.37 for items 29 and 30 to 0.66 for item 31 (All the necessary information for diagnostic and therapeutic decisions is routinely available to me). The data are presented in Table 4.

**Table 4:** Discriminatory power of SAQ-SF scales.

SAQ subscales		Correlation of positions Total	Multiple correlation square	Cronbach’s alpha after removing the item
**Teamwork Climate**	1. Nurse input is well received in this clinical area.	0.367	0.222	0.621
	2. In this clinical area, it is difficult to speak up if I perceive a problem with patient care.	0.221	0.071	0.685
	3. Disagreements in this clinical area are resolved appropriately (i.e., not *who* is right, but *what* is best for the patient).	0.440	0.235	0.595
	4. I have the support I need from other personnel to care for patients.	0.411	0.298	0.610
	5. It is easy for personnel here to ask questions when there is something that they do not understand.	0.536	0.388	0.564
	6. The physicians and nurses here work together as a well-coordinated team.	0.406	0.244	0.606
**Safety Climate**	7. I would feel safe being treated here as a patient.	0.560	0.379	0.685
	8. Medical errors are handled appropriately in this clinical area.	0.640	0.465	0.661
	9. I know the proper channels to direct questions regarding patient safety in this clinical area.	0.555	0.421	0.686
	10. I receive appropriate feedback about my performance.	0.622	0.436	0.667
	11. In this clinical area, it is difficult to discuss errors.	0.023	0.024	0.819
	12. I am encouraged by my colleagues to report any patient safety concerns I may have.	0.463	0.311	0.704
	13. The culture in this clinical area makes it easy to learn from the errors of others.	0.487	0.299	0.700
**Job Satisfaction**	15. I like my job.	0.371	0.164	0.840
	16. Working here is like being part of a large family.	0.644	0.440	0.771
	17. This is a good place to work.	0.719	0.525	0.747
	18. I am proud to work in this clinical area.	0.728	0.537	0.746
	19. Morale in this clinical area is high.	0.593	0.404	0.787
**Stress Recognition**	20. When my workload becomes excessive, my performance is impaired.	0.714	0.616	0.809
	21. I am less effective at work when fatigued.	0.749	0.638	0.795
	22. I am more likely to make errors in tense or hostile situations.	0.692	0.497	0.819
	23. Fatigue impairs my performance during emergency situations (e.g. emergency resuscitation, seizure).	0.651	0.466	0.841
**Perceptions of Management**	24. Management supports my daily efforts: Unit /Mgt	0.849	0.731	0.936
	24. Management supports my daily efforts: Hosp /Mgt	0.818	0.689	0.918
	25. Management doesn’t knowingly compromise Pt safety: Unit /Mgt	0.768	0.632	0.950
	25. Management doesn’t knowingly compromise Pt safety: Hosp/ Mgt	0.731	0.575	0.932
	26. Management is doing a good job: Unit /Mgt	0.854	0.743	0.910
	26. Management is doing a good job: Hosp /Mgt	0.900	0.814	0.927
	27. Problem personnel are dealt with constructively by our: Unit /Mgt	0.885	0.824	0.930
	27. Problem personnel are dealt with constructively by our: Hosp /Mgt	0.864	0.783	0.908
	28. I get adequate, timely info about events that might affect my work, from: Unit /Mgt	0.880	0.800	0.931
	28. I get adequate, timely info about events that might affect my work, from: Hosp /Mgt	0.840	0.749	0.913
**Working Conditions**	29. The levels of staffing in this clinical area are sufficient to handle the number of patients.	0.372	0.149	0.815
	30. This hospital does a good job of training new personnel.	0.372	0.149	0.815
	31. All the necessary information for diagnostic and therapeutic decisions is routinely available to me.	0.659	0.474	0.637
	32. Trainees in my discipline are adequately supervised.	0.579	0.411	0.682

* Hosp–hospital; Mgt-management, Pt- patient.

Notes: The discriminatory power of particular entries of SAQ-SF scales ranged from 0.02 for item 11 to 0.90 for item 26.

## Discussion

The analysis of the matrix of the main components obtained in our study revealed that out of six main components separated from the eight, the highest factor loadings belonged to the questions measuring the same dimensions of safety climate, according to the original scale [13]. The first and the second components were defined by coefficients measuring the Perception of the Hospital and Unit Management, respectively while on the first component there were the factors related to the Hospital Management by the board, and on the second–to the Unit Management. The third component included items measuring Climate and Safety of Teamwork. The fourth component grouped items related to Job Satisfaction. The fifth component was defined by elements connected with Stress Recognition, while the sixth gathered the items measuring Working Conditions. The seventh component included the items measuring Climate and Safety of Teamwork. The eighth component grouped the items reversely worded, possibly reflecting the challenges of accurate translations of reverse-scoring items when it is not culturally common to use that measurement technique. The process of cultural adaptation of the SAQ-SF resulted in a reliable and valid Polish version.

The factor structure of the answers as well as tables of adjustment concerning factor loadings of statements were analysed. Item 12 had the lowest loading of position coefficient, i.e. 0.38. Also item 13 had a low loading of position coefficient equal 0.39. The loading of position coefficient for the other items ranged from 0.44 to 0.90. Slightly different results were obtained by Danish investigators [16]–only one item had a coefficient loading of 0.28; the factor explained less than 8% of variances. In the research of Kristensen et al., item 28 had a low loading of position coefficient 0.33. In Kristensen’s research all the other elements had a position coefficient load between 0.41 and 0.88. Question No. 3 has its highest loading on the same component as items 4 and 5; on the 7^th^ component it occurs quite high, where questions 1 and 6 are contained. Question 12 also indicates the highest loading on the same component as questions being components of the Safety Climate and Teamwork Climate sub-scale.

It is worth noticing that in our study item No. 3, related to the resolving of disagreements and settlement of disputes, and question No. 12, regarding concerns about patient’s safety, were also on the component where questions characterising the sub-scale of perception of the hospital management were found, while according to the original version they were situated among the components describing Teamwork Climate and Safety. This finding may result from the conviction of medical staff–in this case mostly nurses–that a conflict should be resolved at the managerial level and any discrepancies should be reported to supervisors. It suggests that interprofessional samples may influence factor loadings, a methods issue for further research.

It is also not possible to strictly compare the obtained results with other adaptations of SAQ-SF because most of them present results of confirmatory factor analysis (CFA) only, which is used to estimate the goodness of fit of theoretical model or previously established factor structure to a new dataset, whereas exploratory factor analysis (EFA) is usually used to estimate factor structure of the newly created tool. Zimmermann et al. obtained good fit of the model for the 34 item SAQ on German data, however it was done after exclusion of five items from the analysis due to low I-CVI index values [35]. They identified a few items from Perception of Management (item 27), Working Conditions (item 29) and Teamwork Climate (item 6) with to low factor loadings. Similarly, Kristensen et al., who obtained good fit of the CFA model for Danish data, also indicated one item (item 2) which had to small factor loadings to define the same subscale as it was stated by the authors of the scale, and another one (item 28) which only slightly exceeded threshold required by the authors [16], however they used very liberal level of 0.3 as suggested by Hair [36]. According to the results presented by Bulajić M. et al in Croatian version of SAQ item 29 occurred to correlate with Perception of Management (like in the original US version) not with Working Conditions [19].

In Poland, there is still a hierarchical structure of working in health care institutions. It is rare for physicians, nurses, physiotherapists, and managing staff to apply consultative decision-making process because authoritarian, functional and organisational structures and mechanisms prevail as the operational culture. Emphasis is placed on strengthening the authority of the management. Discipline and order are the most important issues and are reinforced by the management structure supporting the maintenance of status quo [37]. This contextual factor may explain some of the differences in factor loadings.

It also has to be noted that in Poland, building a safe organisational culture in healthcare facilities requires many various features and behaviours of the staff. The most important of them include responsibility for one’s own actions and actions of co-workers which might influence patients’ safety by reporting adverse events or actions posing a threat to patient’s safety to the internal errors committee rather than awareness of the whole health care team oriented towards safety patient centered care climate [38].

Amongst other published adaptations of the instrument, the reliability of subscales of the Polish SAQ-SF version ranged from 0.74 to 0.95, and only for Teamwork Climate Cronbach alpha equalled 0.66. In most countries which adapted SAQ-SF, there was at least one subscale that did not reach a satisfactory level of reliability. Table 5 compares the Polish results to other countries [7, 14, 16–19, 22, 24–26, 35, 39].

**Table 5:** Results Polish version of the SAQ-SF compared with other countries.

Countries	Cronbach’s α					
	Teamwork Climate	Safety Climate	Job Satisfaction	Stress Recognition	Perceptions of Management	Working Conditions
**Poland**	0.66	0.74	0.82	0.86	0.93–0.95	0.75
**The Netherlands** [7]	0.76	0.77	0.84	0.69	0.65	0.57
**Albania** [14]	0.79	0.82	0.78	0.62	0.64	0.76
**Denmark** [16]	0.70	0.76	0.84	0.78	0.86	0.72
**Taiwan** [17]	0.79	0.82	0.91	-	0.87	0.78
**China** [18]	0.78	0.82	0.90	0.88	0.88	0.78
**Croatia** [19]	0.59	0.66	0.84	0.79	0.77–0.80	0.75
**Italy** [22]	0.73	0.72	0.83	0.78	0.84–0.86	0.70
**Brazil** [24]	0.65	0.67	0.77	0.78	0.75–0.79	0.65
**Slovenia** [25]	0.58	0.76	0.79	-	0.76	-
**Sweden** [26]	0.81	0.75	0.89	0.86	0.72	0.72
**Switzerland** [35]	0.65	0.75	0.79	0.79	0.44	0.65
**USA** [39]	0.80	0.83	0.88	0.71	0.65	0.71

Notes: Amongst other published adaptations of the instrument, the reliability of subscales of the Polish SAQ-SF version ranged from 0.74 to 0.95, and only for Teamwork Climate Cronbach alpha equalled 0.66. In most countries which adapted SAQ-SF, there was at least one subscale that did not reach a satisfactory level of reliability.

Nonetheless, given healthcare system and cultural differences between countries, factors loading differently are not necessarily a problem and may simply reflect the local context of healthcare [40]. What is important is that the concepts comprising the factor loadings remained consistent in our study, even if the items loaded on different subscales.

### Limitations and implications of research

The study has several limitations. First, the trial of this study is limited to one hospital in Poland, which raises questions about the possibility of generalizing the results. Secondly, the research sample is rather small, but close to the minimum size of the group. Third, the collected data came mainly from nurses and physicians. The results of the representatives of other medical professions accounted for about 15%. Given the limitations, future research should extend these results to more hospitals in Poland. The accuracy of the scale should also be tested by comparing different groups of medical professions, and their selection would take into account a comparable number of respondents.

## Conclusions

The Polish version of the SAQ-SF questionnaire meets the psychometric and functional validation criteria and presents satisfying psychometric properties (validity and reliability). The SAQ-SF-PL version is an instrument which will allow a valid and reliable assessment of Safety Climate in healthcare facilities, such as hospitals, thus enabling identification of areas requiring improvement or support as well as allowing comparison with other international studies. The instrument can be used to generate data to inform management’s policies and actions to improve patient safety.

## Supporting information

S1 File Postawy wobec bezpieczeństwa: Perspektywy personelu pracującego w danym obszarze opieki nad pacjentem (SAQ-SF PL). (PDF)
