# Supplementary material for: Cultural adaptation of the Safety Attitudes Questionnaire – Short Form (SAQ-SF) in Poland
Source: PLoS One. 2021 Feb 5;16(2):e0246340. doi: 10.1371/journal.pone.0246340 (PMC7864443; doi:10.1371/journal.pone.0246340)
Supplement: S1 File — (PDF) [file pone.0246340.s001.pdf]

Postawy wobec bezpieczeństwa: Perspektywy personelu pracującego w danym obszarze opieki nad pacjentem (SAQ-SF PL)

Pracuję w (proszę określić obszar kliniczny lub obszar opieki nad pacjentem, w którym spędza Pan/Pani najwięcej czasu) ______

Jest to oddział: ______ Prosimy o wypełnienie ankiety w oparciu o własne doświadczenia w tym oddziale szpitalnym.

USE A NO. 2 PENCIL ONLY

Poprawnie zaznaczona odpowiedź ⬬ **Niepoprawnie zaznaczona odpowiedź** ✓ ✗ ◒ ◉

Proszę odpowiedzieć na poniższe pytania odnosząc się do oddziału szpitalnego, w którym Pan/Pani pracuje.

Proszę wybrać odpowiedź używając poniższej skali:

A	B	C	D	E	X
Zdecydowanie nie zgadzam się	Raczej się nie zgadzam	Odpowiedź neutralna	Raczej się zgadzam	Zdecydowanie się zgadzam	Nie dotyczy

	Zdecydowanie nie zgadzam się	Raczej się nie zgadzam	Odpowiedź neutralna	Raczej się zgadzam	Zdecydowanie się zgadzam	Nie dotyczy
1.Praca pielęgniarek w moim oddziale jest postrzegana pozytywnie.	A	B	C	D	E	X
2.W tym oddziale trudno jest mówić otwarcie, jeśli dostrzegam problem dotyczący opieki nad pacjentem.	A	B	C	D	E	X
3.Spory pojawiające się w oddziale są właściwie rozstrzygane (tj. z perspektywy tego, *co* jest najlepsze dla pacjenta, a nie tego, kto ma rację).	A	B	C	D	E	X
4.W opiece nad pacjentami otrzymuję od pozostałych członków zespołu wsparcie, którego potrzebuję.	A	B	C	D	E	X
5.Członkowie zespołu nie mają problemów z zadawaniem pytań, jeśli czegoś nie rozumieją.	A	B	C	D	E	X
6.Lekarze i pielęgniarki tworzą dobrze skoordynowany zespół.	A	B	C	D	E	X
7.Czułbym/Czułabym się bezpiecznie, będąc leczony/a tu jako pacjent/pacjentka.	A	B	C	D	E	X
8.W tym oddziale sprawy dotyczące zdarzeń niepożądanych są właściwie prowadzone.	A	B	C	D	E	X
9.Wiem jak i do kogo kierować pytania dotyczące bezpieczeństwa pacjenta na tym oddziale.	A	B	C	D	E	X
10.Otrzymuję odpowiednie informacje zwrotne dotyczące mojej pracy.	A	B	C	D	E	X
11.Na tym oddziale trudno jest dyskutować o błędach.	A	B	C	D	E	X
12.Moi koledzy zachęcają mnie do zgłaszania wszelkich wątpliwości dotyczących bezpieczeństwa pacjenta, jakie mi się nasuwają.	A	B	C	D	E	X
13.Kultura pracy na tym oddziale ułatwia uczenie się na błędach innych.	A	B	C	D	E	X
14.Gdybym zgłosił/zgłosiła swoje sugestie dotyczące bezpieczeństwa kierownictwu, zostałyby one uwzględnione.	A	B	C	D	E	X
15.Lubię moją pracę.	A	B	C	D	E	X
16.Praca tutaj, to jak bycie częścią dużej rodziny.	A	B	C	D	E	X
17.To dobre miejsce do pracy.	A	B	C	D	E	X
18.Jestem dumny/dumna z pracy na tym oddziale.	A	B	C	D	E	X
19.Morale na tym oddziale są wysokie.	A	B	C	D	E	X
20.Kiedy jestem przeciążony/przeciążona pracą, jakość pracy jest niższa.	A	B	C	D	E	X
21.Jestem mniej efektywny/efektywna w pracy, kiedy jestem zmęczony/zmęczona.	A	B	C	D	E	X
22.W sytuacji napięcia lub wrogości ryzyko, że popełnię błąd jest większe.	A	B	C	D	E	X
23.Zmęczenie ma negatywny wpływ na moją pracę w nagłych przypadkach (np. reanimacja, atak padaczki).	A	B	C	D	E	X
24.Kierownictwo wspiera mnie w codziennych wysiłkach*: kier. oddziału A B C D E X dyr. szpitala	A	B	C	D	E	X
25.Kierownictwo nie naraża świadomie bezpieczeństwa pacjentów*: kier. oddziału A B C D E X dyr. szpitala	A	B	C	D	E	X
26.Zarządzający wykonuje dobrze pracę*: kier. oddziału A B C D E X dyr. szpitala	A	B	C	D	E	X
27.Problemy personelu są rozwiązywane konstruktywnie przez*: kier. oddziału A B C D E X dyr. szpitala	A	B	C	D	E	X
28.Otrzymuję terminowo odpowiednie informacje, które mogą mieć wpływ na moją pracę od*: kier. oddziału A B C D E X dyr. szpitala	A	B	C	D	E	X
29.Poziom zatrudnienia personelu w oddziale jest odpowiedni w stosunku do liczby pacjentów.	A	B	C	D	E	X
30.Szpital prawidłowo szkoli nowy personel.	A	B	C	D	E	X
31.Mam standardowo dostęp do wszystkich informacji niezbędnych do podejmowania decyzji diagnostycznych lub terapeutycznych.	A	B	C	D	E	X
32.Osoby szkolące się w moim zawodzie są odpowiednio nadzorowane.	A	B	C	D	E	X
33.Moje doświadczenia współpracy z pielęgniarkami na tym oddziale są dobre.	A	B	C	D	E	X
34.Moje doświadczenia współpracy z lekarzami na tym oddziale są dobre.	A	B	C	D	E	X
35.Moje doświadczenia współpracy z farmaceutami na tym oddziale są dobre.	A	B	C	D	E	X
36.Często występują zaburzenia komunikacji, prowadzące do opóźnień w zapewnieniu opieki.	A	B	C	D	E	X

INFORMACJE DODATKOWE

Czy wypełniał/a Pan/Pani tę ankietę wcześniej? ○ Tak ○ Nie ○ Nie wiem **Dzisiejsza data (miesiąc/rok):**__________________

Zajmowane stanowisko: (Proszę zaznaczyć tylko jedną odpowiedź):

- ○ Lekarz ordynator/Lekarz etatowy
- ○ Lekarz specjalista na stażu naukowym
- ○ Lekarz rezydent
- ○ Lekarz/pielęgniarka z tytułem magistra
- ○ Pielęgniarka oddziałowa/ zarządzająca
- ○ Dyplomowana pielęgniarka
- ○ Farmaceuta
- ○ Terapeuta (fizjoterapeuta, ter. zajęciowy)
- ○ Kliniczny pracownik socjalny
- ○ Dietetyk
- ○ Prac.wsparcia med.** (np. opiekun med., ratow. med.,itd.)
- ○ Technolog/technik (zabiegowy, laboratoryjny, radiologiczny,itd.)
- ○ Pracownik wsparcia admin. (urzędnik/prac. sekretariatu, itd.)
- ○ Wsparcie środowiskowe (sanitariuszka, prac. firmy sprz., itd.)
- ○ Pozostałe funkcje kierownicze (np. kierownik kliniki)
- ○ Inne:..

Proszę zaznaczyć płeć: ○ Mężczyzna ○ Kobieta **Przeważająca grupa pacjentów:** ○ Dorośli ○ Dzieci ○ Obie grupy

Doświadczenie zawodowe: ○ Poniżej 6 m-cy ○ 6 do11 m-cy ○ 1 do 2 lat ○ 3 do 4 lat ○ 5 do10 lat ○ 11 do 20 lat ○ 21 lat i więcej

Dziękujemy za wypełnienie ankiety – jesteśmy wdzięczni za udział w badaniu i poświęcony nam czas.

PROSZĘ NIE WYPEŁNIAĆ

○ ○

* W pytaniach od 24 do 28 proszę o wyrażanie opinii zarówno w odniesieniu do kierownika oddziału jak i dyrektora szpitala.

** Proszę o zakreślenie zajmowanego stanowiska pracy.

2017 Iwona Malinowska-Lipień, Uniwersytet Jagielloński-Collegium Medicum za zgodą The University of Texas at Austin
